# Forensic odontology identification response to terrorist attacks in Paris November 2015

**DOI:** 10.1080/20961790.2020.1778847

**Published:** 2020-11-02

**Authors:** Steve Toupenay, Aida Ben Cheikh, Bertrand Ludes, Rufino Felizardo

**Affiliations:** a UFR Odontologie, Université de Paris, Paris, France;; b Université de Paris, BABEL, CNRS, Institut Médico-Légal de Paris, Paris, France

**Keywords:** Forensic sciences, forensic odontology, terrorist attack, forensic identification, disaster victim identification

## Abstract

The terrorist attacks of November 2015 led to the immediate death of 129 victims admitted to the Legal and Forensic Medicine Institute of Paris, including 41 unidentified. During the Disaster Victim Identification (DVI) operations, 22 bodies were examined by the postmortem (PM) dental team with the aim of establishing PM odontograms. At the same time, the dental expert in the antemortem (AM) unit collected a large number of dental files, progressively filtered as the list of missing persons became reduced. Feedback from these events has highlighted the difficulties of implementing the DVI chain principles in a legal framework, published the day before the attacks, and also the technical complexity of collecting dental data on a week end of terror. The return on experience after this event has represented a paradigm shift on previous methods of DVI in Paris and even more in France. Indeed, the victim identification procedure was redesigned, integrating new technical means such as a CT scan directly on spot, allowing the extraction of maxillofacial data as soon as possible in order to support the PM dental examination team. Moreover, the National Dental Council proceeded to the overall remodeling of the dental identification unit, which is composed of trained members, from local, regional and national aspects. These forensic experts are dedicated, at the request of the legal authorities, to DVI operations and deployed throughout the country capable of managing AM and PM data. This unit aims also to share experiences and awareness-raising among health professionals and investigators in order to optimize a better submission of AM elements and also to enhance the major interest of odontology as a primary identifier in disaster.

## History of forensic odontology in mass disaster

Like fingerprints or DNA, forensic odontology is considered as one of the three primary identifiers according to international recommendations such as those of International Criminal Police Organization (INTERPOL). These identifiers were implemented to help in the victims identifications of the terrorist attacks occurred on the 13 November 2015, in Seine-Saint-Denis (Stade de France) and in different locations in Paris, France.

Historically, it was in Paris on 1897, that the odontological identification was scientifically applied and codified for the first time in the context of a mass disaster following the fire of the Charity Bazaar, where 121 victims perished mostly carbonized. Among them are many aristocratic Parisian women for whom dentists, such as Davenport for the Duchess of Alençon, came with their clinical dental records in order to compare and formally identify the burned bodies and remains. Subsequently, this use of odontology, put forward by Oscar Amoedo (1898) [1] was widely used during the identification process of victims such as natural disasters (tsunami in the Indian Ocean, 2004), aeronautical crash accidents (Concorde Paris, 2000; AF447 Rio Paris, 2009), and major fires (Australian Bush, 2009; Grenfell Tower in London, 2017).

## In the context of the terrorist attacks

On Friday 13 November 2015, at 21:17, a first suicide attack took place at the gates of the Stade de France in Seine-Saint-Denis killing three terrorists and a passerby. In a simultaneous and coordinated way, at 21:25 started a bloody run through the terraces of restaurants and bars of Paris killing 39 people until one of the terrorists unleashed his explosive belt. Fifteen minutes later, a third team of terrorists entered the Bataclan, concert hall in Paris, where 90 victims were killed during the worst hostage crisis in France. Thus 129 people died that evening from the bullets and explosions of this terrorist attack which represents the most important catastrophe in terms of victims since the Second World War. These attacks lead to a massive influx of victims with large-scale ballistic injuries, given the type of weapons used (kalashnikov), and transported to the Legal and Forensic Medicine Institute of Paris at the request of the judicial authority [2].

In a fortuitous way, the lessons learned the same year from the previous Paris attacks at the office of the newspaper “Charlie Hebdo” in January 2015 as well as the attacks at the Bardo Museum in Tunisia in March 2015, lead the authorities to publish, the day before, a series of interministerial directives on the care of victims of terrorist attacks (Interministerial Directives on the Care of Victims of Terrorism Acts 2015) [3].

The Prosecutor of the Republic of Paris then provided for the requisition and organization of the operations of determining the cause of death and victims identification in close connection with the seized medicolegal teams.

The following day, the bodies of 123 victims and 17 fragments were admitted to the Legal and Forensic Medicine Institute of Paris. Although the majority of the victims had identifiers that are supposed to be related to the circumstances during the body recovery, 41 bodies were recorded as unidentified (under X). Within 2 h after mobilization of the Legal and Forensic Medicine Institute of Paris, the director set up the forensic odontology team supported by four forensic odontology experts attached to the forensic institute as well as one expert of the Criminal Investigation Institute of the National Gendarmerie (Institut de recherche criminelle de la gendarmerie nationale, IRCGN). Members of this team were experienced in mass disaster identification and had already been involved in international missions. They remained at the disposal of the authorities and available 24/7. One of them was mobilized on the antemortem (AM) team, the four others in the postmortem (PM) team. The communication between both teams then passed through the police authorities. These practitioners were under the command of the Chief of the Forensic Division and the operational units of the Scientific and Technical Police who was in charge of the Disaster Victim Identification (DVI) operations.

This article of Paris terrorist attacks is the first original report providing forensic odontology data and analysis.

## The PM dental data collection

The thanatological operations to determine the identity and the cause of death began quickly (external examinations and autopsies). All bodies admitted to the forensic medicine institute benefited from a two-dimensional (2D) digital radiological examination including frontal and lateral radiographs of the skull and neck, the thorax, the abdomen-pelvis, upper and lower limbs in search of prostheses, fractures and dental elements. The facial and profile images of the skull and neck allowed people to know whether or not there are identifying dental elements, such as dental care, prostheses, implants, osteosynthesis plates or orthodontic appliances. However, it was not possible to determine either the nature or the exact position due to the superimpositions between the left and right sides.

The team of PM odontologists was mobilized to carry out the odontological examinations on the bodies registered as “unidentified”. Due to the external examinations and autopsies at first, the PM dental examinations were carried out 24 h later.

The forensic experts applied the PM odontological examination protocols according to INTERPOL recommendations (INTERPOL, 2018) [4] and established a PM odontogram of the unidentified victims using the DVI INTERPOL form for Missing Person, dedicated to PM examinations (tab 600) [5].

The records should include:

- Manual documentation of odontology done on the principle of double examination to enhance scientific validity [4].
- Intra-oral radiographs: bite wings (molars from both sides jaws in contact) retro-alveolar X-rays of molars, premolars and incisors in particular teeth with dental care, prosthesis or specific morphology. The intra-oral digital images were made using a conventional radiovisiography (RVG) generator available at the Paris Forensic Institute and supported by a second portable generator from the IRCGN. The quality of the records were evaluated in terms of exposure, density and sharpness [4].
- Dental photographs were taken by an investigator from the Police and recorded by a PM identification number. The images should include extra-oral photographs, with frontal and profile views, and intra-oral photographs, full mouth with close up images of upper and lower dental arches, frontal and lateral views with teeth in contact and lips retracted and photographs of teeth with specific anatomy, dental care, prosthesis, fracture or anomalies.

All the PM dental records were included in the PM data of the unidentified victims files.

## The AM dental data collection

At the same time, the Police Prefecture of Paris provided a call number available for families and relatives of missing persons. The Public Information Centre recorded more than 1 700 callers. The first list of missing persons was based on these data.

In the AM team, a single expert in forensic odontology supported the collection of AM dental data which was initially installed in the Police Prefecture before being transferred to the Ministry of Interior. This odontologist worked closely with the investigators who provided him with an AM list of missing persons with a dentist or identifiable dental elements.

The exhaustive list of suspected disappearances resulted in a significant loss of time since it was reduced from hour to hour but mistakenly triggering the search for AM documents while the person was still alive. The desperate families, without any news of their loved ones, first went around the hospitals before finishing the search for a loved one at the forensic institute of Paris. Before being redirected to the Ecole Militaire where the AM cell for the families of victims was set up, this massive influx to the reception of the forensic institute led to a delay for the investigators who were unable to contact families looking for different information on the missing person such as the contact details of the dentist.

The dental expert was then in charge of calling the practitioners of the missing persons in order to collect the original dental records necessary for the writing of an AM odontogram. The expert applied the AM protocol according to INTERPOL recommendations, AM form DVI INTERPOL Missing Person [6], dedicated to AM examinations (tab 600).

The search for dental identifying elements should include (INTERPOL, 2018):

- Detailed dental care sheet: tooth number (according to the World Dental Federation notation), treatment nature, location, type of material, equipment (fixed, implant, removable, orthodontic, etc.)
- Radiographic images: in 2D (retroalveolar, bitwings, orthopantomogram, lateral cephalogram, etc.) and/or in three-dimensional (3D, eg. CBCT, scanner, etc.)
- Photographs: extra-oral and intra-oral images (orthodontic, surgical, prosthetic or social media photographs such as selfies)
- Additional elements: plaster models and casts; orthodontic appliances (removable brace, retainers, etc.); removable dentures; splints; letters of correspondence, discharge; financial notice; etc.

All the documents were centralized on a single and secured electronic address. These data must be processed and sorted downstream by the DVI police officers before reaching the odontologist who is then responsible for assessing the quality of the data, acknowledging the receipt to the dentist contacted and sometimes asks for a supplement or a new transmission with better image quality, which results in many time-consuming steps.

In addition, the AM dental data collection suffered from the combination of different elements: the fact of being at weekend with many closed practices, the difficulty in contacting practitioners on their mobile phones which falls under the private domain. The main difficulty was to obtain the contact details of the treating practitioner from the files of the Primary Health Insurance and supplementary insurance when the families did not have information. In the absence of practitioner contact detail, it was attempted in some cases, depending on the address of the missing victim, to establish a list of practitioners by areas in order to call the offices in search of AM documents.

AM files were progressively edited and manually written with the clinical files, the data from the 2 D and 3 D radiographs, the photographs as well as the elements of the orthodontic file, taking into account the population of young adults. AM dental records were included in the AM data of the missing victims files.

## Conciliation of the AM and PM dental records: results

The conciliation aimed to match PM data with AM data with the view to identify the victims. All documents and images had been anonymized according to ethical policy for patient data research.

### PM examination

**Figure 1.**
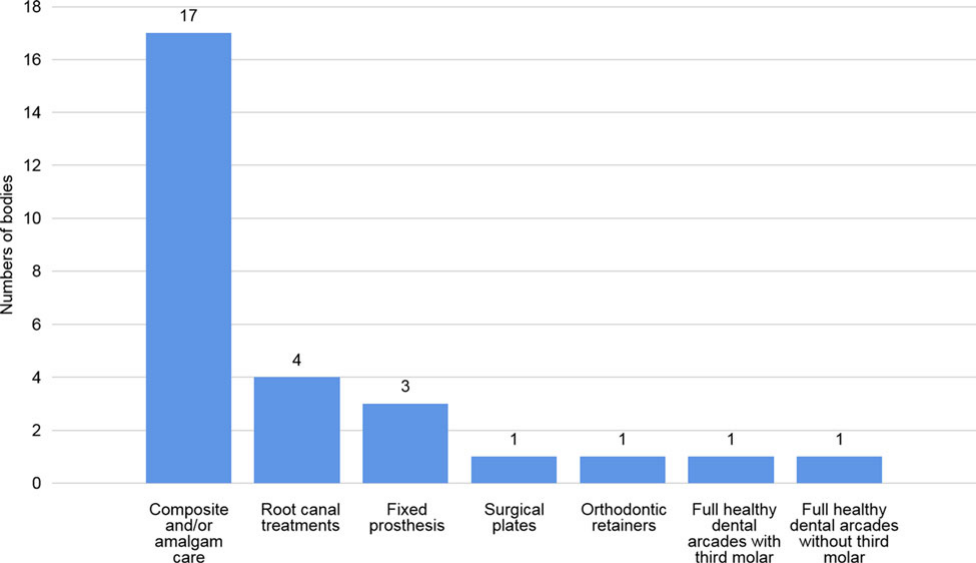
Postmortem (PM) dental elements identifiers on examined bodies (22 PM odontograms).

The PM odontological expertise began on the afternoon of Saturday, November 14, 2015. Of the 41 bodies registered as unidentified, 22 PM odontograms were performed. The PM identifying elements found were of various nature and location. The majority of dental examinations revealed the presence of composite and/or amalgam care, which was consistent with the most common care in the general population, especially among young adults. Root canal treatments, fixed prosthesis and orthodontic retainers were observed. Two odontograms with healthy teeth were established with and without wisdom teeth (DDS). The location, the nature of the materials, the type of orthodontic retainers, tooth morphologies, as well as the stage of root edification of wisdom teeth were important elements to be noted during the PM examination (Figure 1).

**Figure 2.**
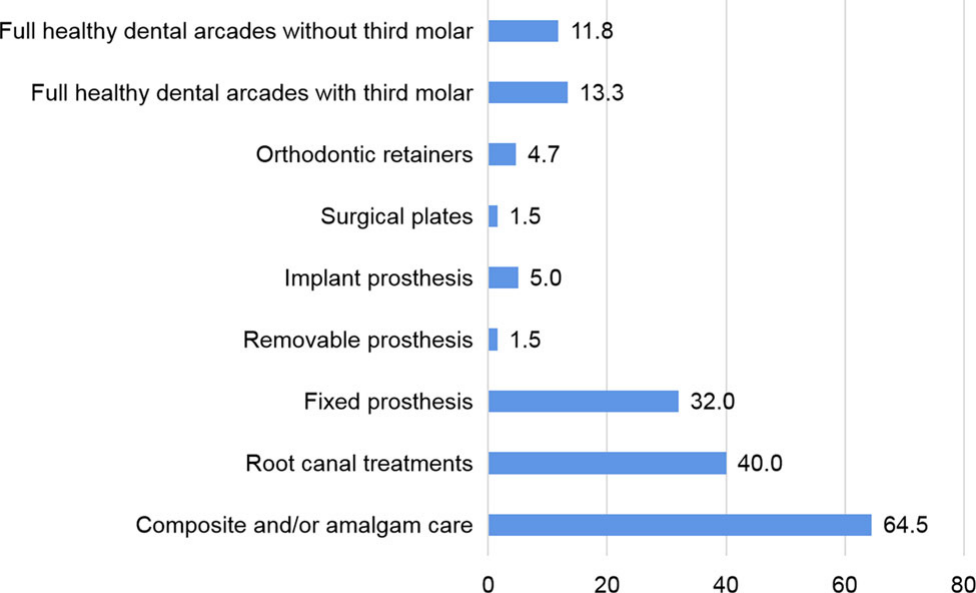
Postmortem dental elements identifiers (%) detected on the 2 D head X-rays performed in all the victims.

The 2D digital radiological examination including frontal and lateral views of the skull and neck were retrospectively analysed in all the bodies admitted in the forensic institute during the attacks. This important work allowed the PM records and identification of the different types of dental elements that could have been helpful during the identification process such as dental care, root canal treatment, fixed or removable prostheses, implants, surgical plates or orthodontic appliances. It is interesting to highlight that 64.5% of victims had dental elements such as composite and/or amalgam care; 40% had root canal treatment and 32% had fixed prosthesis; 4.7% had orthodontic retainers (Figure 2).

### AM data collection

The forensic odontologist began the data collection as soon as the AM list was received, on Saturday 14 November, 2015 in the early afternoon. The exhaustive list of missing persons and the lack of contact details for practitioners during the weekend contributed to a more laborious collection of information. However, the first AM records were quickly formed after receiving the information through the investigators. Of the 30 AM files searched, 24 AM odontograms were completed on the Tuesday morning of 17 November, and 88% of contacted practitioners sent the AM files.

**Figure 3.**
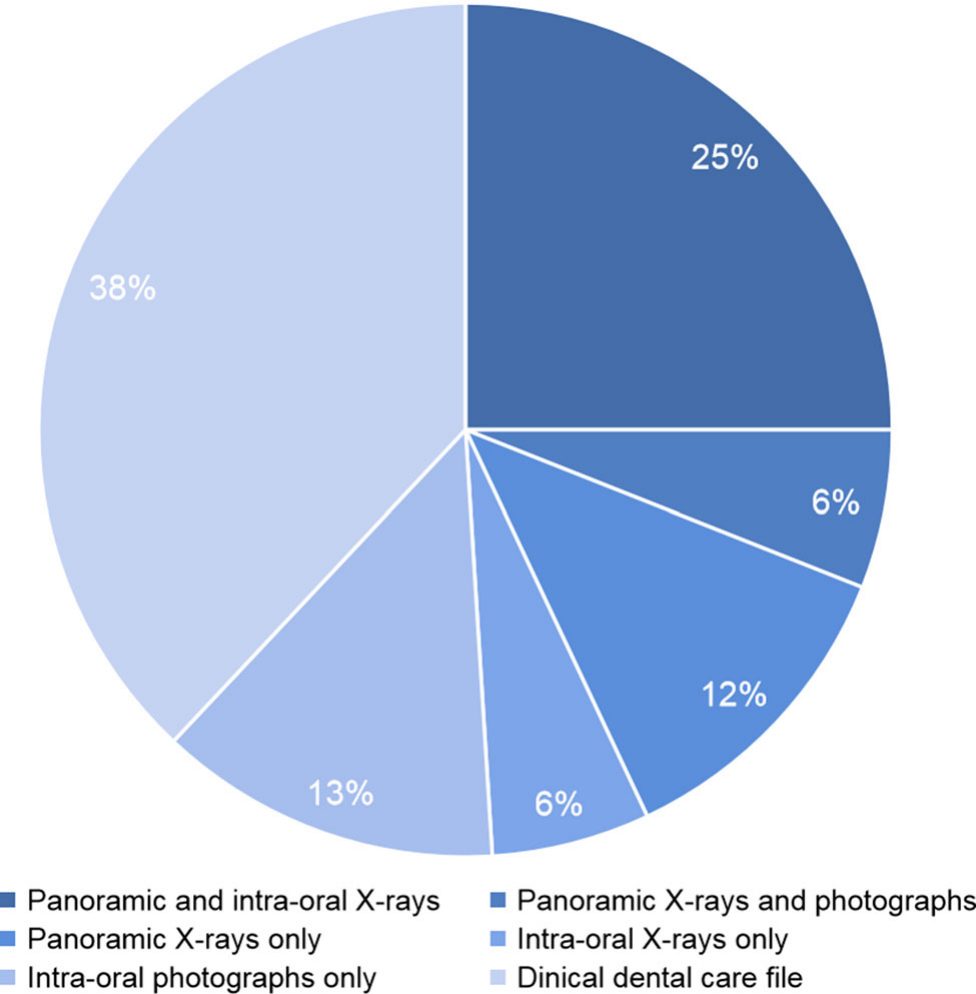
Antemortem files components.

Most of the AM dental records consisted of radiographic and photographic documents (Figure 3). They were all analyzed in order to identify and fill the AM odontogram for each missing person.

The identifying dental elements collected in the AM data were of various natures. The elements found in the documents were: amalgam and composite care; endodontic treatments; fixed crowns (metal and ceramic); dental implants; osteosynthesis plates from orthognathic surgery Lefort 1; dyschromias with enamel defects and hypomineralization of molars and incisors; third molars included, extracted or absent; orthodontic appliances and different orthodontic retainers (removable or fixed). It is important to notice and report all details in the AM odontograms.

### AM and PM conciliation

**Figure 4.**
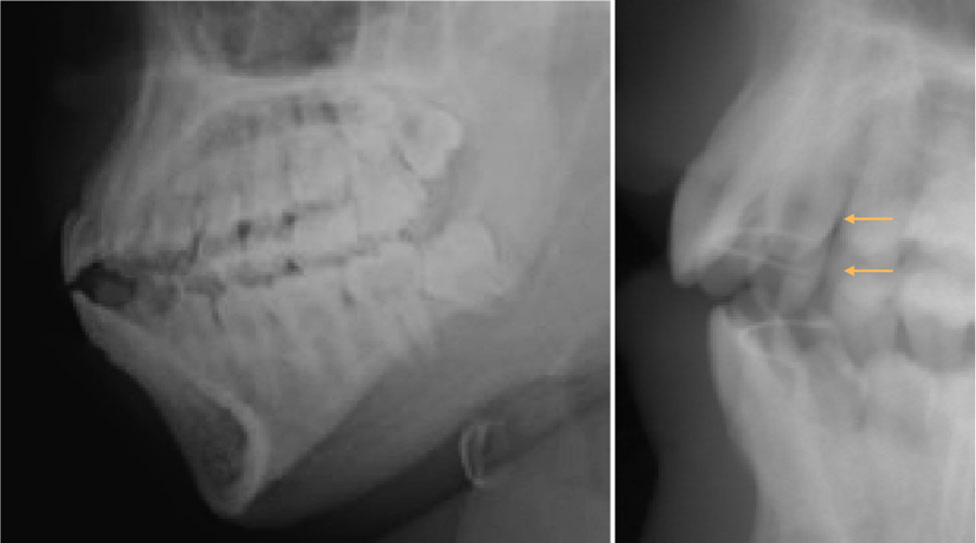
First-line postmortem 2 D radiographs, no dental care, complete arches few (mostly morphometric) data. On the right, there are only two orthodontic retainers (arrows).

**Figure 5.**
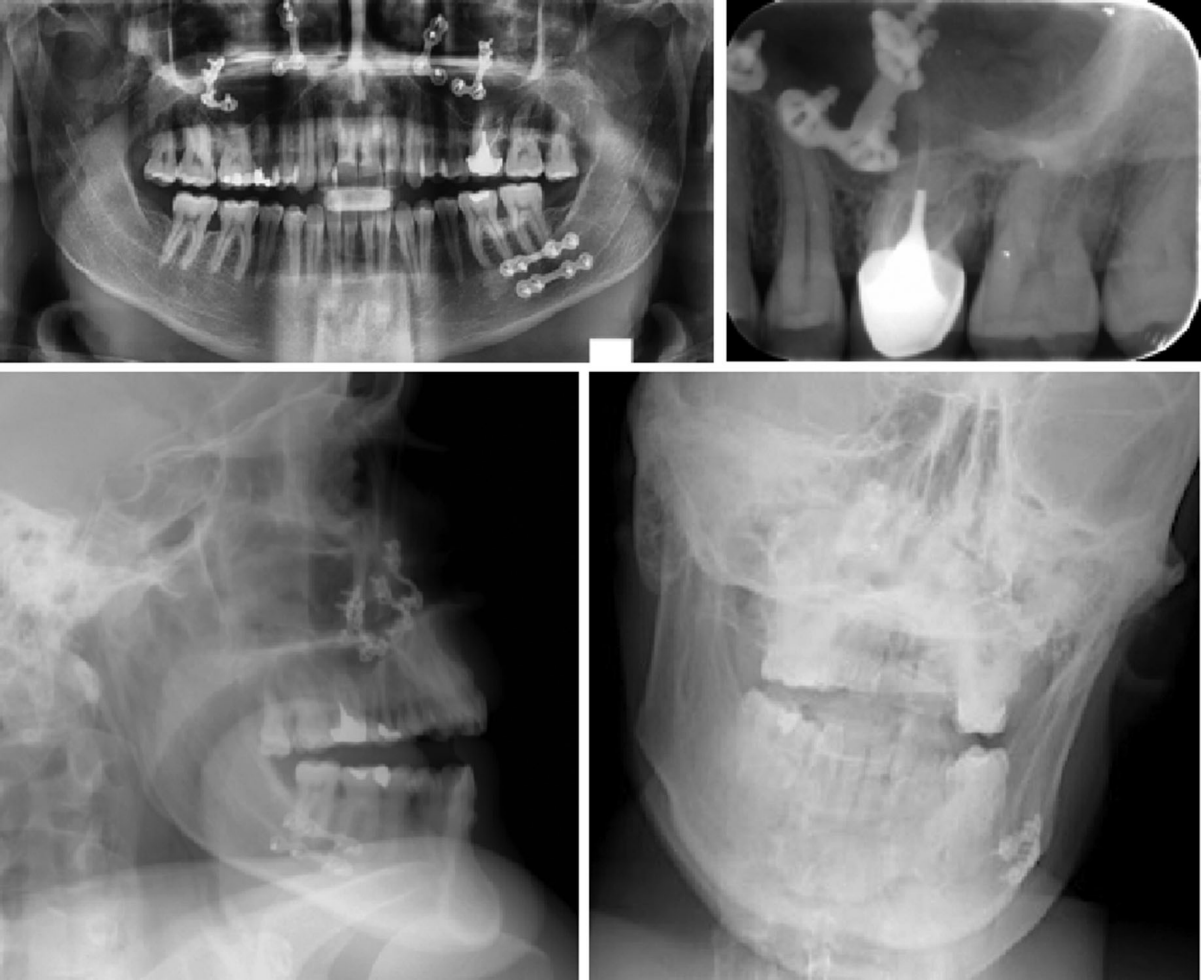
Antemortem (top) and postmortem (bottom) with orthognathic surgery osteosynthesis material.

**Figure 6.**
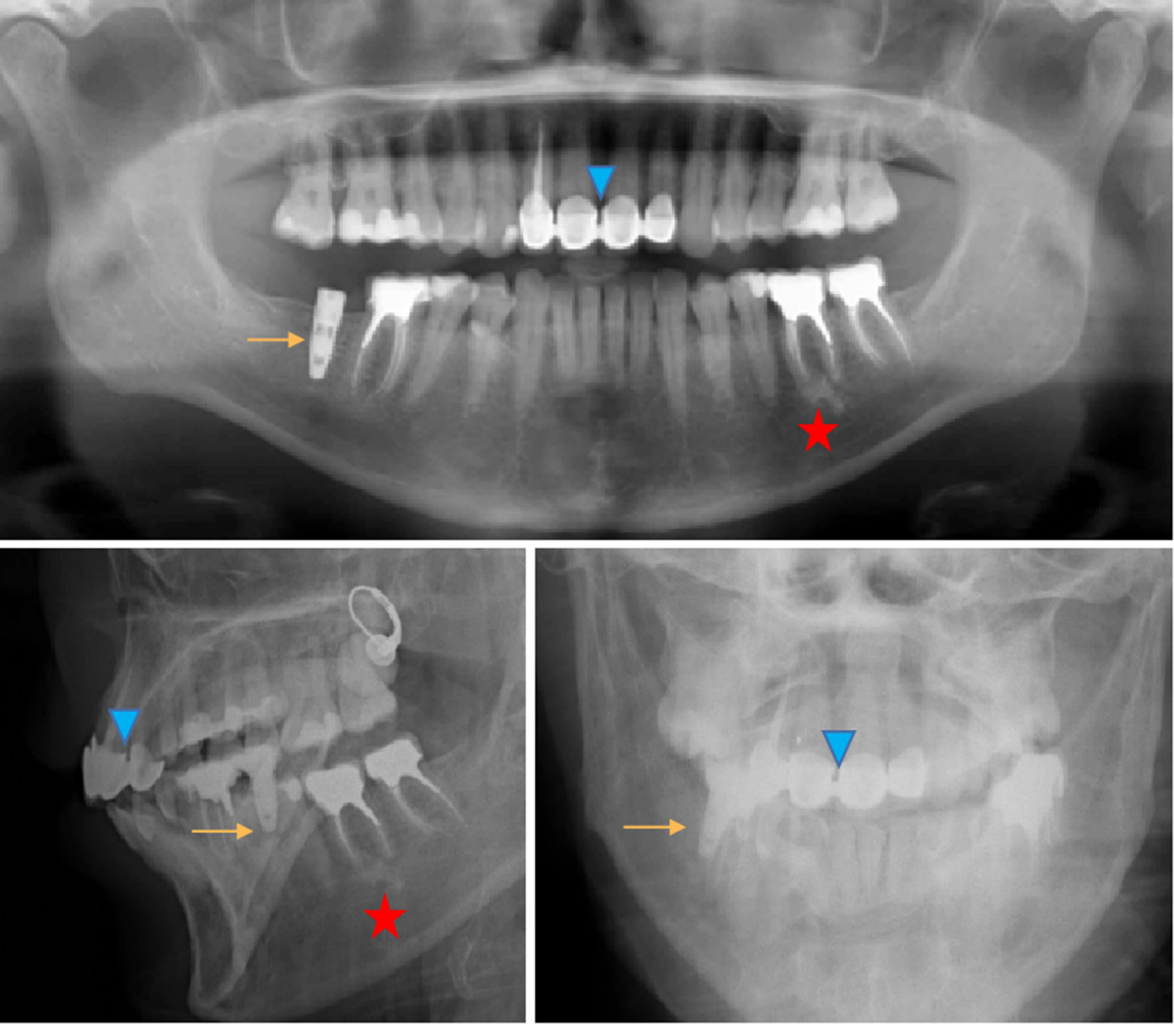
Antemortem radiological record (top) and 2 D postmortem face and profile images (bottom) with several strong markers: implant (arrow), anterior bridge (arrowhead), apical cementosis (star).

**Figure 7.**
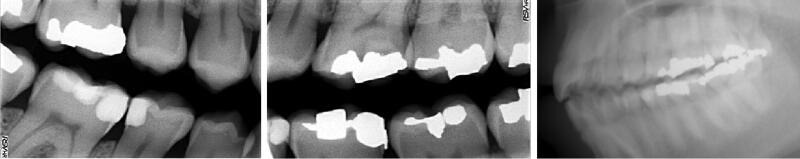
Antemortem X-ray record by bite-wings images, numerous amalgam and composite fillings (left and middle), postmortem image of first intention (right) superimposition of coronary radiopaque images, in this case intra-oral images are indispensable.

These elements can be important discriminating clues in the comparison of AM and PM elements necessary for formal identification (Figures 4–7).

The commission for the identification of the victims of the disaster met on the morning of 23 November. The comparison of AM and PM odontological data led to establishing seven formal identifications purely dental and one formal identification both dental and fingerprint, which were primary identifiers elements.

## Challenges faced

### Context related

The psychological impact and the massive influx of victims in a short time did not allow the implementation of interministerial directives, regarding the care of victims of terrorist attacks, published the day before the terrorist attacks. Therefore the recommendations could not benefit from upstream sharing and training of the different teams in case of mass disaster.

The doctrine of thanatological operations for the identification and determination of causes of death delayed the dental examination for bodies registered as unidentified. A large majority of victims were admitted with presumed identities sometimes requiring a posteriori rebuttal of the identity and a new registration of the body as unidentified.

### Radiological 3D images/equipment related

At that time, the odontological team could not systematically benefit from extended radiological data in the absence of on-site 3 D scanning as well as 2 D extra-oral images which were sometimes taken after odontological examination. Only intra-oral X-rays allowed the collection of dento-osseous information.

### AM data collection related

The opening of a nominative missing persons list triggered the search for AM data which were sometimes carried out unnecessarily. In some cases, families forgot to warn the authorities when the loved one was found alive while AM investigations were still ongoing.

Another difficulty encountered in AM was the lack of rapid information gathering. Having a young man on a Friday evening in a concert hall sometimes caused a delay. In fact, families were not aware of the his social life. Therefore, they could not solicit and inform the AM cell about the disappearance, neither did they know the details regarding the dental practitioner treating the young adult, living alone in Paris, away from his parents’ home. In addition, the young generation, strongly affected during these terrorist attacks, presents less large-scale dental care thanks to the preventive actions. However, a significant number of young adults have or had an orthodontic treatment with braces or retainers that allow full records collection with photographs, radiographs, dental casts and documents provided by the orthodontist, who is most often known to parents.

Regarding the dental profession, the main difficulty was to contact practitioners during the weekend in a particular context generated by terrorist attacks. Despite the immediate mobilization of the information technology (IT) department and the administrative teams of the National Dental Council, the private mobile phone numbers of the practitioners were not informed, the latter were therefore not reachable before the opening of the practices on Monday. Many of the practitioners’ records from the Dental Council were incomplete without a direct number that could be used to respond to a medical or forensic emergency.

All data were sent by emails and centralized on a single and protected address which delayed the entry of AM dental data.

### Conciliation related

The number of odontologists engaged in the requisitions by the authority was limited and did not allow for fluidity in the management of AM and PM dental data that did not take place at the same time. A small number of PM dental examinations on the total number of victims was achieved by four experts in forensic odontologists, and the AM data collection were performed on several potential victims from the large list of missing persons by one forensic odontologist.

The absence of a clearly identified coordinator within the odontologists under command of the DVI police units required the transmission of AM and PM data through them and not directly from practitioner to practitioner.

The exchanges were carried out using paper documents and through the scientific and technical police units of the DVI, avoiding e-mail exchanges.

AM and PM data of primary identifiers, including fingerprints, DNA and odontology, were collected by one person in the forensic institute of Paris. This coordinator was in charge of preparing the comparison of these elements before the identification committee of conciliation that met on 23 November.

## Return on experience—paradigm shifts

The feedback from the events that occurred in November 2015 in Paris represented a paradigm shift on previous methods of DVI protocol in Paris. These events reinforced certain specific points in the text of the Interministerial Directives, which was adopted in November 2017 [7].

Indeed, in contrast to the decision made in November 2015 during the identification process, victims who cannot decline their identity because they are unconscious or dead must be registered as “unidentified person” or “under X” in French, in the SINUS census database. The old term “X could be” is outlawed because of the confusion generated. All body recovery operations are the exclusive responsibility of the designated investigators. The recovery of a dead person is accompanied by an at-the-scene examination report that identifies precisely all the elements found near the victim and likely to contribute to the identification.

The DVI process was reviewed and highlighted the importance of prioritizing the identification of the victims at first. Since then, a sequential operation is integrated in three clearly identified areas. After registration of the body, the process included acquisition of radiological data of the deceased by conventional “whole body” CT scan, followed by forensic external records by the investigators of the scientific and technical police in DVI, fingerprinting; DNA and odontological examination prior to medicolegal forensic operations.

The CT scan is therefore integrated into the process in the interministerial directives of 10 November 2017, but remains at the discretion of the Public Prosecutor. This step was integrated into the DVI protocol of the Legal and Forensic Medicine Institute of Paris, where the simulation exercise in real conditions in October 2018 enabled the observers of the Paris Public Prosecutor’s Office and the Paris Police Prefecture to be aware of the advantages of these types of imaging.

**Figure 8.**
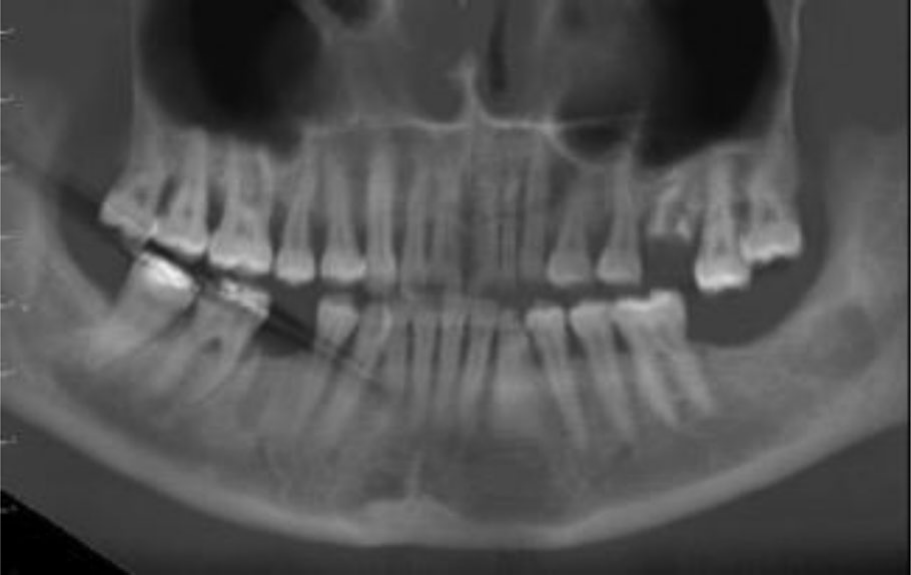
Panoramic reconstructed in thick section from a CT scan “head bone” volume.

**Figure 9.**
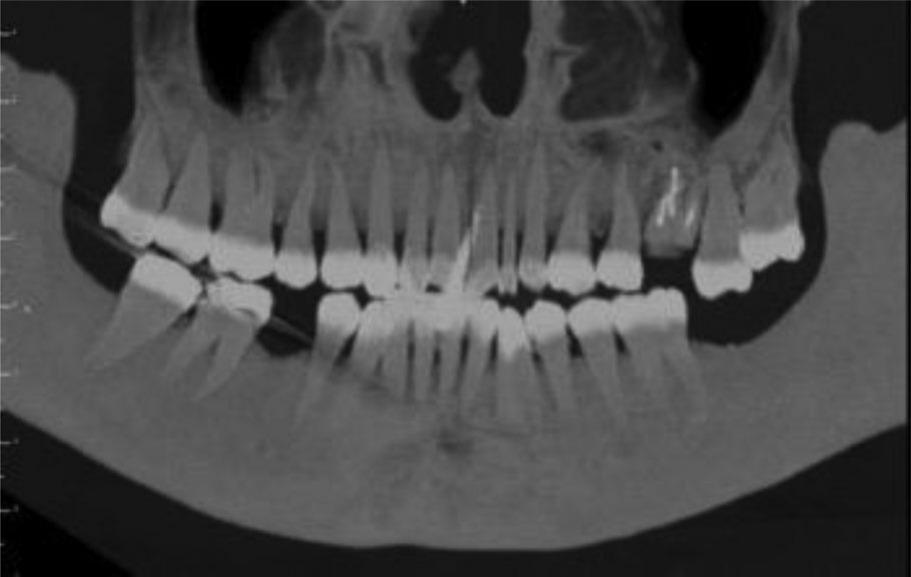
Panoramic view in maximum intensity projection mode.

**Figure 10.**
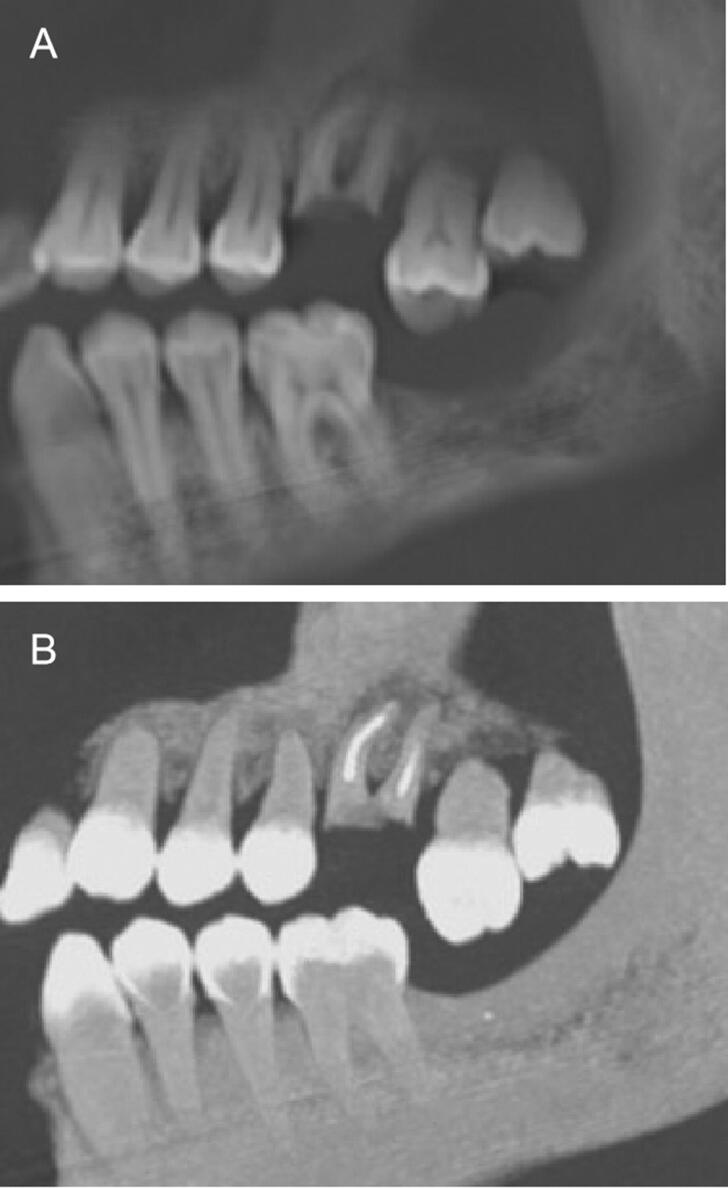
(A) Left side, first maxillary molar in the form of roots with endodontic treatment-egression second molar. (B) Maximum intensity projection mode in idem.

An odontologist specifically dedicated to medical imaging is in charge, which ensures the linkage between the CT scan process and the odontological examination. Separating the data of the dento-maxillofacial area from the CT scan volume of the head allows specific reconstructions of interest to odontologists engaged in a DVI process (Figures 8–10). They benefit instantaneously from a radiological overview of the dental arches as well as an odontogram based on radiographic data (blue in colour) according to an internally established protocol, validated and already used in Paris DVI situation (fire disaster) in 2019.

In parallel but very often offset from the time of the odontological PM examination of the victim, the radiological or clinical data are collected by the AM unit.

Here, the dental volumetric data take all their interest. Indeed, it is a posteriori possible to perform any mulitplanar or sagittal oblique reconstruction mimicking any AM radiological data. This flexibility in the manipulation of the images confers a dynamic view to the examination but not a static view as could be a 2 D intra-oral radiographs in the sense that we can navigate within the arches and confirm or refute a hypothesis comparison [8–11]. At the Legal and Forensic Medicine Institute of Paris, the room to perform odontological identification operations on two or three bodies at the same time is near the medical CT scan. To avoid any further manipulation, the body bags are left on the transport trolleys which then serve as examination tables. The teams consist of two odontologists who examine the body and a third who enters the data on the odontogram. PM oral photographic views are made by a photographer of the judicial identity of the Scientific and Technical Police.

At the end of these operations dental identification file is complete and contains: classic odontogram after direct examination, radiological digital odontogram and key images reformated from the CT scan, and photos of the Judicial Identity.

These elements are transmitted to the dental coordinator expert who will be present at the identification committee. Unlike 2015, there is now a coordinator for each primary identifier (DNA, Dactyloscopy, Odontology).

In the interest of the DVI operation and INTERPOL recommendations, the forensic experts are now also trained on an international DVI software, known as the Plass Data system [12], which records, through computer processing, both AM and PM data on two separate INTERPOL IT forms. Plass Data allows quicker matches based on comparison points and also permits the standardization of the elements. Indeed, the importance of international standardization is even more relevant as terrorist attacks usually occur on touristic areas, requiring more time during the DVI process if two different terminologies are used in the AM and PM data for the same dental element. To alleviate this problem, the standardization of the terminology for forensic oro-dental data is ongoing by the International Organization for Standardization (ISO/DIS 20888) [13].

The dental care professionals and first and foremost the French National Dental Council has engaged material resources and reorganized its task force in forensic odontology. The “Dental Identification Unit” (UIO) brings together 65 odontologists trained in forensic odontology, mostly experts in Court. The current number is spread over the national territory, departments, regions and overseas communities (DROM COM) in order to allow a quick mobilization following a disaster. These practitioners undergo annual training and have specific equipment including a mobile X-ray generator. All clinical disciplines are represented, including dentofacial orthodontics, regarding age of victims as it was the case during the attack on 14 July 2016 in Nice [14]

The difficulties encountered in AM to contact practitioners are being resolved since it is necessary to provide an emergency contact number (private telephone number), in the event of a health or forensic crisis, which is requested during the annual registration of professionals in the Dental Council. The involvement of the French National Dental Council as well as its departmental councils are complete in the updating of the practitioners’ files and the sensitization of these by a communication campaign supported by the articles of professional newsletter.

In order to avoid saturation of the hospital sites or the Legal and Forensic Medicine Institute of Paris, a single location for the reception of families and the centralization of AM data is provided for in the DVI plan (Health Emergencies Crisis Management Centre (HECMC)). An AM team, from the odontological identification unit, is seconded to it and is able to establish the AM odontograms resulting from the synthesis of the radiological and clinical data of the dental file provided by the practitioners of the victims as well as the data coming from the health insurance. These organizations now have emergency numbers to facilitate contacts for the collection of AM administrative data.

## Conclusion

In the face of these terrible catastrophes in terms of human accounting, the odontological teams integrate the DVI missions in synergy with other teams. In the same way as the identification by genetic or digital fingerprints, the odontological characteristics of the victims are primary identifiers for which it is essential to be able to ensure a rapid response.

As painful as it may be, each disaster is an opportunity for having feedbacks in order to improve the protocols. The DVI mission of November 2015 has contributed to a paradigm shift on previous DVI protocol in Paris and even more in France. These changes have led to the restructuration of protocols for PM examinations by trained members of a task force, the integration of new imaging techniques (reconstructions from CT scan data and X-rays mobile units) tested during DVI training in real conditions and used for dental identification on a daily basis and during mass disaster. Paris attacks also initiated the remodeling of the Forensic Odontology team from local, regional and national aspects through the UIO, from the French National Dental Council. This has contributed to the improvement and implementation of new process regarding equipment and training, the sensitization of forensic odontology, the improvement of AM data collection through general dentist practitioners.

Since these attacks, all of the changes and the Interministerial Directives, adopted in November 2017, highlight the importance of the identification protocol in mass disaster in France. These improvements are made in order to allow not only families, rightsholders but also the whole society to face disaster and identify victims with the greatest respect for Human Rights.
